# *GRB10* rs1800504 Polymorphism Is Associated With the Risk of Coronary Heart Disease in Patients With Type 2 Diabetes Mellitus

**DOI:** 10.3389/fcvm.2021.728976

**Published:** 2021-09-28

**Authors:** Yang Yang, Wentao Qiu, Qian Meng, Mouze Liu, Weijie Lin, Haikui Yang, Ruiqi Wang, Jiamei Dong, Ningning Yuan, Zhiling Zhou, Fazhong He

**Affiliations:** ^1^Department of Pharmacy, Zhuhai People's Hospital (Zhuhai Hospital Affiliated With Jinan University), Zhuhai, China; ^2^College of Pharmacy, Jinan University, Guangzhou, China; ^3^Department of Pharmacy, The Second Xiangya Hospital, Central South University, Changsha, China

**Keywords:** type 2 diabetes mellitus, *GRB10*, genetic variation, vascular complications, lipid metabolism

## Abstract

Diabetic vascular complications are one of the main causes of death and disability. Previous studies have reported that genetic variation is associated with diabetic vascular complications. In this study, we aimed to investigate the association between *GRB10* polymorphisms and susceptibility to type 2 diabetes mellitus (T2DM) vascular complications. Eight single nucleotide polymorphisms (SNPs) in the *GRB10* gene were genotyped by MassARRAY system and 934 patients with type 2 diabetes mellitus (T2DM) were included for investigation. We found that *GRB10* rs1800504 CC+CT genotypes were significantly associated with increased risk of coronary heart disease (CHD) compared with TT genotype (OR = 2.24; 95%CI: 1.36–3.70, *p* = 0.002). Consistently, levels of cholesterol (CHOL) (CC+CT vs. TT, 4.44 ± 1.25 vs. 4.10 ± 1.00 mmol/L; *p* = 0.009) and low density lipoprotein cholesterin (LDL-CH) (CC+CT vs. TT, 2.81 ± 1.07 vs. 2.53 ± 0.82 mmol/L; *p* = 0.01) in T2DM patients with TT genotype were significant lower than those of CC+CT genotypes. We further validated in MIHA cell that the total cholesterol (TC) level in *GRB10*-Mut was significantly reduced compared with *GRB10*-WT; *p* = 0.0005. Likewise, the reversed palmitic acid (PA) induced lipid droplet formation in *GRB10*-Mut was more effective than in *GRB10*-WT. These results suggest that rs1800504 of *GRB10* variant may be associated with the blood lipids and then may also related to the risk of CHD in patients with T2DM.

## Introduction

Diabetes is a metabolic disease that is characterized by hyperglycemia. This disease is caused by abnormal glucose, lipid, and protein metabolism. Ultimately, this leads to impaired insulin secretion, insulin resistance, or both. The common types of diabetes include type 1 diabetes mellitus (T1DM), type 2 diabetes mellitus (T2DM), and gestational diabetes. T2DM is caused by ineffective use of insulin by the body. T2DM patients are characterized by high blood sugar, relative lack of insulin, insulin resistance and so on (1). Statistical evidence shows that T2DM accounts for more than 95% of all diabetic cases in the Chinese population (2). Over time, T2DM is becoming an increasingly serious problem for global health. The International Diabetes Federation estimated that there were 463 million adults aged 20–70 years with T2DM worldwide in 2019. By 2045, this number is predicted to increase to 700 million (3). T2DM patients often have various complications, including multi-organ damage caused by macrovascular and microvascular complications (4). Diabetic vascular complications significantly increase the disability and mortality rate of diabetic patients, seriously affect the quality of life and cause a huge national economic burden. In a systematic review of 4,549,481 patients with T2DM, the incidence of macrovascular complications was determined to be 32.2%, of which 21.2% of patients had coronary heart disease (CHD) (5). It is evident the CHD has become the main factor threatening the health and life of patients with T2DM. A previous study reported that the occurrence of T2DM vascular disease showed clear associations with both ethnicity and family history (5, 6). Furthermore, multiple studies have reported that gene polymorphisms are associated with diabetic vascular complications (7–9). Therefore, the identification of susceptibility genes related to T2DM vascular complications could provide a possible treatment strategy for the early prevention of this disease. These factors are of great significance with regards to prolonging the survival period of patients and improving their quality of life.

Growth factor receptor-binding protein 10 (GRB10) is an adaptor protein of the GRB7/GRB10/GRB14 protein family. GRB10 can interact with a variety of tyrosine kinase receptors and affect a variety of signal pathways (10). Furthermore, GRB10 has been confirmed to play an important role in regulating cell proliferation, apoptosis, and metabolism, as well as many signaling pathways (11–13). GRB10 is expressed at high levels in tissues that are involved in insulin action and glucose metabolism, including the muscles, pancreas and fat. The IGF/IGFR signaling pathway plays an important role in the regulation and conduction of diabetes mellitus and related complications. GRB10 can interact with IGFR to regulate the IGF/IGFR signaling pathway in a negative manner (14). In one study, the minor allele (MA) of *GRB10* rs4947710 was associated with a reduced risk of T2DM in white subjects from Italy. In another study, *GRB10* rs2237457 genetic variation was associated with T2DM in the Amish population (15, 16). These studies proved that *GRB10* gene polymorphism is closely related to susceptibility for T2DM. As we all know, vascular endothelial growth factor (VEGF) is an important regulator of angiogenesis and it is involved in the development and progression of many angiogenesis dependent diseases. According to previous reports, GRB10 could be involved in a positive feedback loop in VEGF signaling. VEGF could stimulate GRB10 expression, and GRB10 overexpression induced an increase in the amount and the tyrosine phosphorylation of VEGF-R2 (17, 18). Furthermore, *GRB10*, as a key downstream mediator of vascular smooth muscle cell (*VSMC*) miR-504 function, is closely related to vascular diseases under the conditions of diabetes mellitus (19). Collectively, this information indicates that *GRB10* may be a key gene involved in the regulation of diabetes mellitus and related vascular complications.

We previously found that GRB10 is highly expressed in cardioembolic stroke patients by using the Gene Expression Omnibus (GEO) database analysis. Furthermore, previous studies have confirmed that GRB10 is closely related to T2DM and vascular diseases (15, 16, 19). Nevertheless, no study has investigated the relationship between *GRB10* gene polymorphism and T2DM cardiovascular complications. In this study, we studied the influence of *GRB10* gene polymorphism on cardiovascular complications in patients with T2DM.

## Patients And Methods

### Diagnostic Criteria

According to the Chinese Guidelines for the Prevention and Treatment of Type 2 Diabetes (2020 Edition), the diagnostic criteria for diabetes are typical diabetes symptoms plus random blood glucose ≥ 11.1 mmol/L; Or add fasting blood glucose ≥ 7.0mmol/L; Or add OGTT 2h blood sugar ≥ 11.1mmol/L; Or add HbA1C≥6.5%. Typical diabetes symptoms include polydipsia, polyuria, polyphagia and unexplained weight loss. Excluding T1DM and special types of diabetes, the patients with T2DM were included in the study. At the early stage of disease, it is sometimes difficult to determine the type of diabetes. If the classification cannot be determined immediately, a temporary classification can be carried out to guide the treatment. Then, according to the patients' initial response to treatment and the clinical manifestations during follow-up, the patients were re-evaluated and classified (20).

Coronary atherosclerotic heart disease refers to heart disease caused by myocardial ischemia, hypoxia or necrosis due to stenosis or occlusion of lumen caused by coronary atherosclerosis, which is referred to as coronary heart disease (CHD) for short (21). The diagnostic criteria of patients with CHD in this study were implemented in accordance with Chinese Guidelines for Clinical Diagnosis and Treatment of Coronary Heart Disease (2010 Edition) (22).

According to the Guidelines for Primary Diagnosis and Treatment of Hypertension in China (2019 Edition), without using antihypertensive drugs, the blood pressure should be measured three times on different days, with systolic blood pressure (SBP) ≥140 mmHg and/or diastolic blood pressure (DBP) ≥90 mmHg. SBP ≥ 140 mmHg and DBP <90 mmHg are simple systolic hypertension. The patient has a history of hypertension, and is currently taking antihypertensive drugs. Although the blood pressure is lower than 140/90 mmHg, he or she is still diagnosed as hypertension (23).

### Patients

This was a retrospective study involving patients with T2DM. The experimental design was approved by the ethics committee of the Institute of Clinical Pharmacology, Central South University and was registered at http://www.chictr.org.cn (Registration number: ChiCTR1800015661). Participants in this study were randomly enrolled from inpatients at the Second Xiangya Hospital of Central South University between December 2017 and December 2019. Our study included patients who were diagnosed with T2DM on admission and aged between 18 and 80 years. We collected clinical data for all participants, including gender, age, height, weight, body mass index (BMI), waist circumference, hip circumference, waist-to-hip ratio, systolic blood pressure (SBP), diastolic blood pressure (DBP), cardiovascular history, cerebrovascular history, smoking history, drinking history, hypertension, hyperglycemia, family diabetes history, triglyceride (TG) level, cholesterol (CHOL) level, high density lipoprotein-cholesterol (HDL-CH) level, low density lipoprotein-cholesterol (LDL-CH) level, and fasting blood glucose level. We also recorded the levels of GLU-60, GLU-120, glycosylated hemoglobin (HbA1C), 25-hydroxyvitamin D, Cpst-0, Cpst-60, Cpst-120, along with the glomerular filtration rate (eGFR) and medication status (hypoglycemic drugs, lipid-lowering drugs, antihypertensive drugs). The clinical endpoint events included type 2 diabetes vascular complications, blood glucose, blood pressure, TG, CHOL, HDL-CH, and LDL-CH. We excluded patients whose discharge diagnosis was not T2DM and those from which the blood samples were not obtained. We also excluded patients who did not provide informed consent and those did not have a complete set of clinical data.

### Candidate Genes and SNP Selection

We identified two gene chip datasets (GSE22255 and GSE58294) relating to cardioembolic stroke patients in the GEO database (Supplementary Table 1). We merged the two raw datasets and applied RMA standardization. Next, we used the R-Limma package to process differential gene analysis, |log_2_FC| > 1.5 and *p* < 0.05 were considered to be statistically significant. Then, we used the ENCODE database to assess the potential function of SNPs within or near candidate genes. We included candidate SNPs with a minor allele frequency ≥ 5% and a pairwise linkage disequilibrium (*r*^2^) < 0.30 within the same and adjacent genes [1,000 Genomes phase 3 Han Chinese in Beijing (CHB)].

### DNA Extraction and Mass Spectrometry Typing

Peripheral venous blood was collected from all patients who met the inclusion criteria. DNA was extracted from peripheral venous blood using the E.Z.N.A. SQ blood DNA Kit II (Omega Bio-Tek Company, USA). The extracted DNAs were then stored at −80°C wait for analysis. The Sequenom MassARRAY SNP system was used to screen the genotypes of all candidate SNPs (Bioyong Technologies Inc.). Information relating to the probe is shown in Supplementary Table 2. Finally, 5% of the participants were randomly selected for verification by Sanger sequencing.

### Cell Culture and Cell Transfection

An immortalized hepatocyte cell line, MIHA, was cultured in Roswell Park Memorial Institute (RPMI) 1640 Medium supplemented with 10% fetal bovine serum (FBS). Cells were then cultured in a standard humidified incubator at 37°C in a 5% CO_2_ atmosphere.

Plasmids containing an empty vector, and the *GRB10* mutation were subcloned into a lentivirus vector (pHBLV-CMV-MCS-3FLAG-EF1-ZsGreen-T2A-PURO) to construct the recombinant plasmid (Hanheng Biotechnology, Shanghai, China). The *GTB10*-Mut plasmid featured an rs1800504 mutation (allele C to T). The detailed information of the construction of *GRB10* mutant lentiviral vector is shown in the Supplementary Files 2. MIHA cells were transfected with Lentiviral vector using Polybrene (Hanheng Biotechnology,Shanghai, China) according to the manufacturer's instructions.When the expression of GRB10 reached its peak, the cell lines with stable expression were screened by Puromycin.

### Western Blot and Real-Time Polymerase Chain Reaction (PCR)

The protocol used for western blotting was described in previous report (24). The membranes were first probed with GRB10 (#3702) and GAPDH (#5174S) primary antibodies purchased from Cell Signaling Technology (CST). Following incubation, membranes were probed with a horseradish peroxidase (HRP)-labeled anti-rabbit secondary antibody from CST (#2708) (diluted with 5% BSA to 1:1,000). Antibody binding was subsequently detected by an enhanced chemiluminescence detection kit (ECL) (Biosharp Biotechnology, BL520B).

Total RNA was extracted from MIHA cells using an RNA-Quick Purification Kit (ES Science). Complementary DNA (cDNA) was synthesized using ReverTra Ace reverse transcriptase (Novoprotein, E04710A) in accordance with the manufacturer's instructions. Real-time RT-PCR was performed with a SYBR qPCR SuperMix Plus Kit (Novoprotein, E096) on a 7300 plus/Bio-RAD iCycler in accordance with the manufacturer's instructions. The primer sequences were as follows: GRB10, forward, 5′-CGAAACTCACCCGTCCAG-3′; reverse, 5′-GGATTCACAGTGTCGGTTGG-3; β-actin: forward, 5′-CATGTACGTTGCTATCCAGGC-3′; reverse, 5′-CTCCTTAATGTCACGCACGAT-3′. The gene expression levels for each amplicon were calculated by the ΔΔCT method and normalized against β-actin mRNA.

### The Determination of Total Cholesterol and Oil Red Staining

According to the Trinder reaction principle, cholesterol makes 4-aminoantipyrine react with phenol (PAP) to produce a red quinoneimine pigment. Using this strategy, we used a TC kit operating standard (BB-47435) and a Microplate Reader to determine the absorbance of samples at a wavelength of 510 nm.

Oil red O can be used to stain lipids in cells. Positive staining changes the color of fat from orange to red. After 24 h, the cells were washed twice with phosphate buffered saline (PBS) and fixed with 4% paraformaldehyde at room temperature for 30 min. After removing the fixative, the cells were washed twice with distilled water and soaked in 60% isopropanol for 5 min. After discarding the isopropanol, we added freshly prepared ORO stain (ORO stain:distilled water; 3:2) for 10–20 min. The staining solution was then discarded, and the cells were washed with water twice. We then added Mayer hematoxylin staining solution to stain the nuclei for cycler 1–2 min, the staining solution was then washed away and replaced with water so that we could monitor the developing staining effect. Finally, we used distilled water to cover the cells and observed the cells with an EVOS microscope (Life Technologies/Thermo Fisher Scientific, US).

### Statistical Analysis

SPSS Statistical software (version 22.0 for windows, Chicago, IL) was used for statistical analysis and GraphPad Prism version 5 software (GraphPad Prism Software Inc., La Jolla, CA) was used to create figures. Power and sample size calculations software (Version 3.0.43) was used to calculate the sample size required for the study. The *T*-test or the Mann–Whitney *U*-test were used for the statistical analysis of measurement data (mean ± standard deviation), as appropriate. The chi-squared or Fisher's exact test was used to determine whether the gene distribution conformed to the Hardy–Weinberg equilibrium. Logistic regression analysis was used to calculate the odds ratio (OR) and 95% confidence interval (CI) between the *GRB10* single-nucleotide polymorphisms (SNPs) and diabetic vascular complications after adjusting for age, BMI, gender, hypertension, smoking and drinking. Comparison of biochemical indicators variance between the *GRB10* genotype was performed with Univariate Analysis of Variance (ANOVA) after adjusting for potential confounders, such as age, BMI and gender. Linkage disequilibrium (LD) and haplotypes analyses were performed using SHEsis online software (http://analysis.bio-x.cn/myAnalysis.php). *p* < 0.05 (2-tailed) was considered to indicate statistical significance.

## Results

### Patient Characteristics and Genotyping

**Figure 1:**
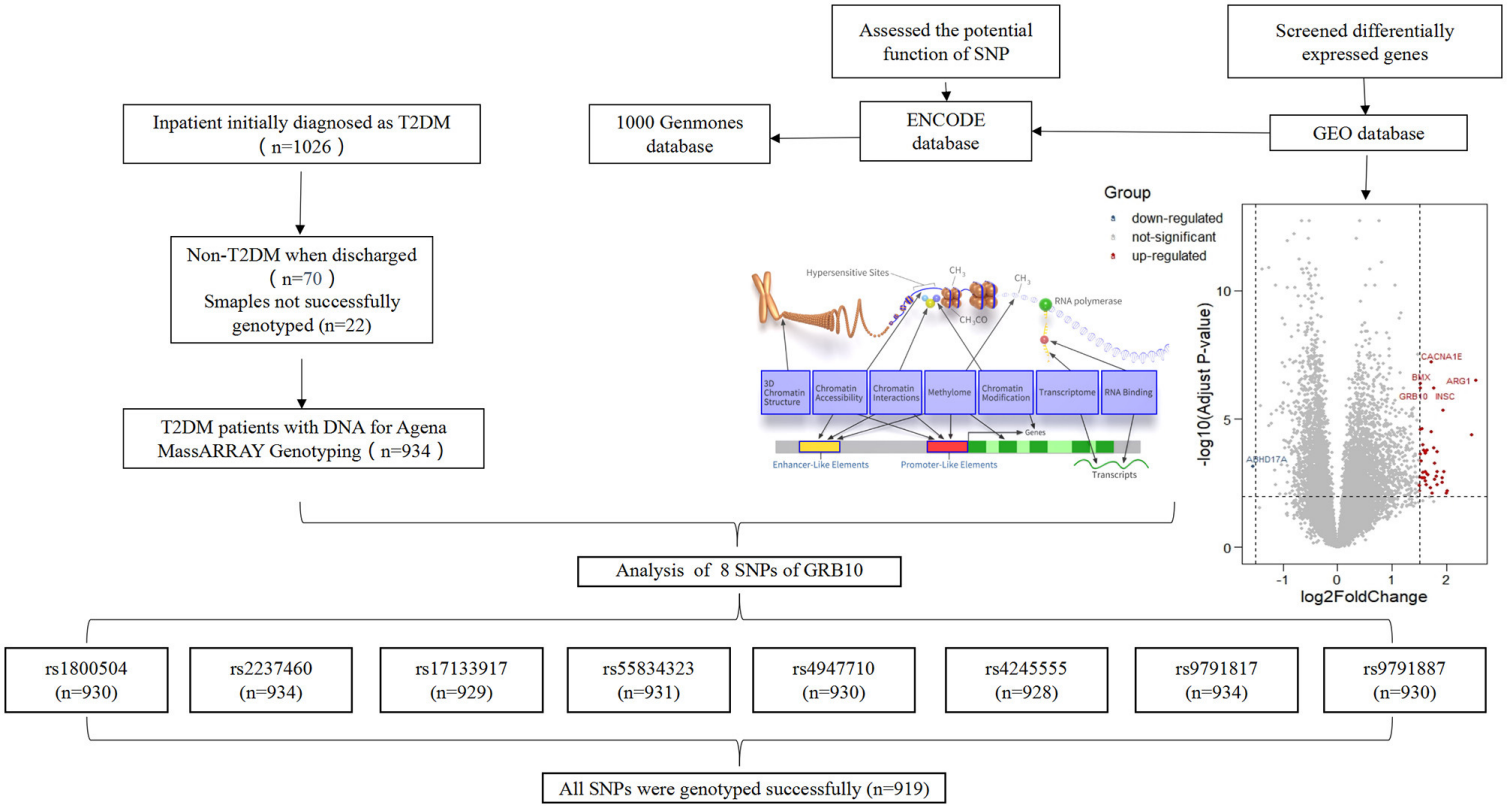
Flow chart showing the research process.

We collected clinical data and matched DNA samples from 1,026 Chinese patients with an initial diagnosis of T2DM. Following final diagnosis, 70 cases were classified as non-T2DM, and 22 DNA samples were not genotyped. Finally, 934 patients were included in the study. According to our research scheme, eight candidate SNPs in *GRB10* were screened, including rs1800504, rs2237460, rs17133917, rs55834323, rs4947710, rs4245555, rs9791817, and rs9791887 (The SNP genotyping data has been uploaded to the DRYAD, https://datadryad.org/stash/share/pDGln0dQK5R8YEMJKAZLJEFs6xWtE7xOi8rD9frUSYA). In total, 919 patients were successfully genotyped with all SNPs (Figure 1). The detailed results related to the loci of these SNPs are shown in Supplementary Table 3. Analysis showed that rs9791887, rs9791817, and rs424555, were not consist with the Hardy-Weinberg Equilibrium. Next, we analyzed the SNP linkage disequilibrium of *GRB10*. Results showed that there was strong linkage among rs9791887-rs4245555, rs9791887-rs9791817, rs55834323-rs4947710, and rs4245555-rs9791817 (Supplementary Figure 1). The baseline clinical characteristics of participants with rs1800504 are shown in Table 1. There were significant differences in BMI; *p* = 0.02.

**Table 1:** Baseline characteristics of T2DM patients with rs1800504.

**Variable**	**Coronary heart disease**		**Non-coronary heart disease**		**Total**	
	**CC+CT**	**TT**	**CC+CT**	**TT**	**CC+CT**	**TT**
	**(*n* = 222)**	**(*n* = 26)**	**(*n* = 542)**	**(*n* = 134)**	**(*n* = 770)**	**(*n* = 160)**
Age (year), mean (SD)	64.6 (9.51)	67.6 (8.25)	56.0 (11.3)	57.8 (10.3)	58.5 (11.5)	59.4 (10.6)
Male, *n* (%)	124 (55.9)	14 (53.8)	323 (59.6)	78 (58.2)	451 (58.6)	90 (56.3)
Smoking history, *n* (%)	72 (36.0)	10 (40.0)	189 (37.1)	40 (31.5)	264 (37.0)	50 (32.9)
Drinking history, *n* (%)	63 (31.7)	7 (29.2)	146 (28.7)	35 (27.3)	211 (29.6)	42 (27.6)
History of cardiovascular disease, *n* (%)	87 (39.7)	14 (56.0)	19 (3.6)	5 (3.8)	106 (14.0)	19 (12.1)
History of cerebrovascular disease, *n* (%)	34 (15.5)	1 (4.0)	37 (6.9)	6 (4.5)	73 (9.6)	7 (4.5)
BMI (kg/m^2^), mean (SD)^*^	24.5 (3.24)	23.7 (3.30)	24.2 (4.30)	23.4 (3.67)	24.3 (4.01)	23.5 (3.60)
Waist-hip ratio, mean (SD)	0.95 (0.07)	0.95 (0.06)	0.96 (0.01)	0.95 (0.37)	0.95 (0.31)	0.93 (0.06)
Vitamin D deficiency rickets, *n* (%)	138 (62.2)	13 (50.0)	352 (64.9)	86 (64.2)	494 (64.2)	99 (61.9)
Fasting blood glucose (mmol/l), mean (SD)	7.36 (2.85)	8.32 (2.31)	7.70 (2.92)	7.52 (2.59)	7.61 (2.91)	7.65 (2.56)
GLU-60 (mmol/L), mean (SD)	11.9 (3.85)	11.2 (3.85)	12.1 (3.74)	10.9 (3.39)	12.0 (3.74)	10.9 (3.42)
GLU-120 (mmol/L), mean (SD)	12.3 (4.07)	13.0 (4.48)	12.3 (4.24)	12.4 (4.09)	12.3 (4.18)	12.5 (4.14)
Cpst-0 (pmol/L), mean (SD)	499.1 (471.2)	440.5 (247.1)	418.8 (322.5)	375.3 (221.1)	441.3 (370.8)	386.0 (225.8)
Cpst-60 (pmol/L), mean (SD)	819.8 (483.0)	772.4 (435.7)	714.0 (531.7)	603.9 (408.5)	748.7 (517.5)	634.9 (412.9)
Cpst-120 (pmol/L), mean (SD)	1,059.5 (840.0)	945.7 (552.2)	920.3 (681.9)	1,100.3 (903.5)	961.1 (733.4)	1,073.4 (852.9)
AST (IU/L), mean (SD)	23.5 (34.4)	19.7 (7.8)	21.6 (15.3)	21.4 (14.2)	22.1 (22.5)	21.1 (13.4)
ALT (IU/L), mean (SD)	23.6 (46.1)	17.0 (6.3)	22.8 (22.1)	22.1 (19.5)	23.0 (30.9)	21.3 (18.2)
CREA (μmol/l), mean (SD)	102.4 (101.2)	82.1 (44.4)	84.3 (77.8)	78.4 (55.7)	89.6 (85.4)	79.0 (53.9)
TBA (μmol/L), mean (SD)	5.5 (5.5)	8.1 (9.1)	5.47 (7.34)	6.15 (7.87)	5.47 (6.80)	6.47 (8.08)
eGFR (ml/min/1.73 m^2^), mean (SD)	80.6 (32.7)	82.8 (40.5)	98.5 (34.2)	96.5 (32.3)	93.7 (34.6)	95.0 (33.3)
**Medical treatment**						
Insulin drugs, *n* (%)	186 (89.9)	21 (95.5)	403 (87.6)	97 (87.4)	594 (88.4)	118 (88.7)
Biguanides, *n* (%)	85 (38.3)	11 (42.3)	267 (49.3)	75 (56.0)	355 (46.1)	86 (53.8)
Insulin agonist, *n* (%)	9 (4.1)	2 (7.7)	34 (6.3)	11 (8.2)	43 (5.6)	13 (8.1)
DPP-4 inhibitor, *n* (%)	91 (41.0)	9 (34.6)	245 (45.2)	51 (38.1)	339 (44.0)	60 (37.5)
GLP-1 receptor agonist, *n* (%)	5 (2.3)	0 (0.0)	19 (3.5)	2 (1.5)	24 (3.1)	2 (1.3)
SGLT-2 inhibitor, *n* (%)	9 (4.1)	0 (0.0)	21 (3.9)	6 (4.5)	32 (4.2)	6 (3.8)
Lipid-lowering agents, *n* (%)	188 (84.7)	21 (80.8)	363 (67.0)	86 (64.2)	556 (72.2)	107 (66.9)
Glucosidase inhibitor, *n* (%)	119 (53.6)	18 (69.2)	305 (56.3)	79 (59.0)	428 (55.6)	97 (60.6)
Calcium Dobesilate, *n* (%)	108 (49.1)	13 (50.0)	253 (46.8)	55 (41.0)	366 (47.7)	68 (42.5)
β-receptor blocker, *n* (%)	81 (36.5)	12 (46.2)	64 (11.8)	13 (9.7)	145 (18.8)	25 (15.6)
Calcium antagonists, *n* (%)	109 (49.1)	11 (42.3)	178 (32.8)	43 (32.1)	290 (37.7)	54 (33.8)
ACEI/ARB, *n* (%)	116 (52.3)	17 (65.4)	198 (36.5)	48 (35.8)	317 (41.2)	65 (40.6)

*BMI, body mass index; GLU, glucose; Cpst, the secretion rate of C-peptide; AST, aspartate aminotransferase; ALT,alanine aminotransferase; CREA, creatinine; TBA, total bile acids; eGFR, glomerular filtration rate; ACEI, angiotensin-converting enzyme inhibitors; ARB, angiotensin receptor blockers;*

* *p-value of any one of Coronary heart disease, Non-coronary heart disease and Total group is < 0.05*.

### The Association Between *GRB10* rs1800504 Gene Polymorphism and Diabetic Coronary Heart Disease in Patients With T2DM

We analyzed the relationships between the eight candidate SNPs in *GRB10* and T2DM cardiovascular complications. We found that rs1800504 genetic variation was significantly related to the occurrence of CHD in T2DM patients; *p* = 0.011. However, there was no significant association with other cardiovascular complications (Table 2). In this study, 930 patients were successfully genotyped for rs1800504. In the recessive model, the CC+CT genotypes were associated with a significantly increased risk of CHD compared with the TT genotype (OR: 2.24; 95% CI: 1.36–3.70; *p* = 0.002; Table 3). Other SNPs had no significant effect on the CHD of T2DM patients (Supplementary Table 4).

**Table 2:** The association between *GRB10* rs1800504 genetic variation and the risk of vascular complications in T2DM patients.

**Events**	**Genotype**	**Number of patients (%)**	**OR (95%CI)**	***p*-value**
Coronary heart disease^*^	TT	26 (16.3)	Ref.	/
	CT	146 (30.6)	2.35 (1.40–3.95)	**0.001**
	CC	76 (25.9)	2.07 (1.18–3.61)	**0.011**
Peripheral neuropathy	TT	101 (63.5)	Ref.	/
	CT	296 (64.5)	1.09 (0.73–1.63)	0.67
	CC	189 (64.5)	1.20 (0.78–1.84)	0.41
Retinopathy	TT	15 (9.4)	Ref.	/
	CT	56 (11.7)	1.06 (0.71–1.57)	0.79
	CC	37 (12.6)	1.20 (0.79–1.83)	0.40
Nephropathy	TT	66 (41.3)	Ref.	/
	CT	282 (47.3)	0.93 (0.62–1.39)	0.72
	CC	122 (41.9)	1.08 (0.70–1.67)	0.73
		33 (11.3)		
Cerebral infarction	TT	17 (10.6)	Ref.	/
	CT	62 (13.0)	1.04 (0.57–1.91)	0.90
	CC	33 (11.3)	1.03 (0.53–2.00)	0.92
Diabetic foot	TT	24 (15.0)	Ref.	/
	CT	79 (16.6)	1.06 (0.63–1.80)	0.82
	CC	49 (16.7)	1.22 (0.70–2.13)	0.49

* *p < 0.05, indicates a significant statistical difference. The bold values mean p value is less than 0.05*.

**Table 3:** The association between rs1800504 and the risk of CHD in T2DM patients.

**Model**	**Genotype**	**Number of patients (%)**		**OR (95%CI)**	***p*-value**
		**CHD (*n* = 248)**	**Non-CHD (*n* = 680)**		
Additive model^*^	TT	26 (16.3)	134 (19.7)	Ref.	/
	CT	146 (30.6)	327 (48.1)	2.35 (1.40–3.95)	**0.001**
	CC	76 (25.9)	215 (31.6)	2.07 (1.18–3.61)	**0.011**
Dominant model	CC	76 (25.9)	215 (31.6)	Ref.	/
	CT+TT	172 (69.4)	461 (67.8)	1.06 (7.38–1.53)	0.74
Recessive model^*^	TT	26 (16.3)	134 (19.7)	Ref.	/
	CC+CT	222 (89.5)	542 (79.7)	2.24 (1.36–3.70)	**0.002**

*CHD, coronary heart disease. Ref., reference*.

* *p < 0.05, indicates a significant statistical difference. The bold values mean p value is less than 0.05*.

### The Relationship Between *GRB10* rs1800504 Gene Polymorphism and Blood Lipid Levels in T2DM Patients

**Figure 2:**
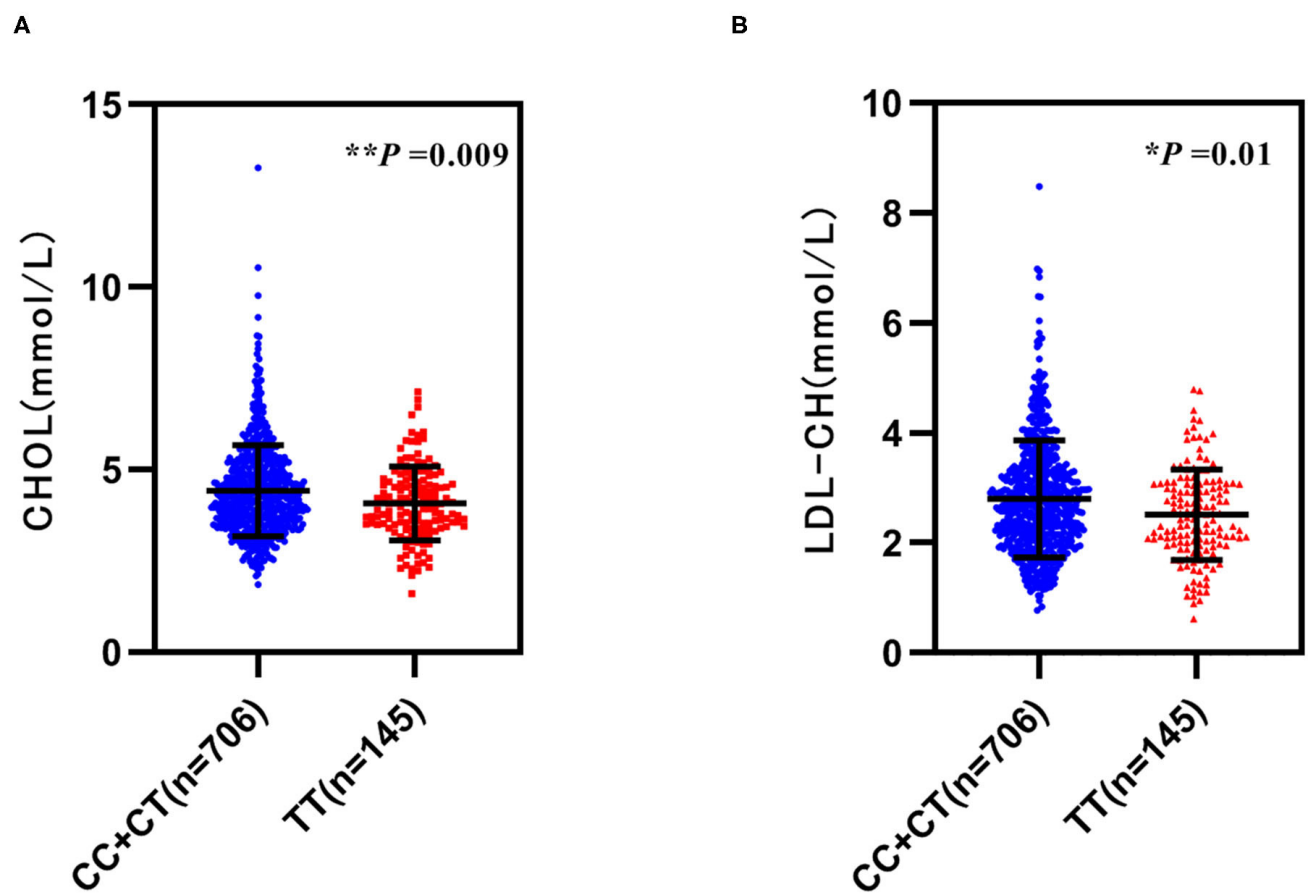
The relationship between *GRB10* rs1800504 gene polymorphism and blood lipid levels inpatients with T2DM. **(A,B)** The levels of cholesterol (CHOL) and low-density lipoprotein (LDL-CH) in a recessive model of rs1800504. Significant differences between groups are indicated by *p < 0.05 and **p < 0.01.

Moreover, we analyzed the relationship between *GRB10* rs1800504 genetic variation and biochemical indicators. We found that rs1800504 variation was associated with plasma lipids level differences in T2DM patients. There were significant differences in the levels of CHOL and LDL-CH when compared between different rs1800504 genotypes. The levels of CHOL in the TT and CC+CT genotypes were 4.10 ± 1.00 mmol/L and 4.44 ± 1.25 mmol/L, respectively; *p* = 0.009. The levels of LDL-CH for the TT and CC+CT genotypes were 2.53 ± 0.82 mmol/L and 2.81 ± 1.07 mmol/L, respectively; *p* = 0.01 (Figure 2 and Table 4). However, none of significant effect was observed as for *GRB10* rs1800504 mutation on blood glucose or blood pressure. Haplotype analysis also showed that the haplotypes of these SNPs did not have a significant impact on the relevant clinical endpoint events (Supplementary Table 5).

**Table 4:** Relationship between rs1800504 and blood lipids in T2DM patients.

**Variable**	**Recessive model**		***p-*value**
	**CC+CT (*n* = 706)**	**TT (*n* = 145)**	
SBP (mmHg), mean (SD)	136.4 (19.8)	135.5 (20.3)	0.71
DBP (mmHg), mean (SD)	80.8 (12.6)	81.2 (13.9)	0.39
Glycated hemoglobin (%), mean (SD)	8.62 (1.99)	8.39 (2.06)	0.26
TG (mmol/L), mean (SD)	2.16 (2.34)	1.94 (1.93)	0.52
CHOL (mmol/L), mean (SD)^*^	4.44 (1.25)	4.10 (1.00)	**0.009**
HDL-CH (mmol/L), mean (SD)	1.03 (0.29)	1.03 (0.29)	0.57
LDL-CH (mmol/L), mean (SD)^*^	2.81 (1.07)	2.53 (0.82)	**0.01**

*SBP, systolic blood pressure; DBP, Diastolic blood pressure; TG, triglyceride; CHOL, cholesterol; HDL-CH, high density lipoprotein-cholesterol; LDL-CH, low density lipoprotein-cholesterol;*

* *p < 0.05, indicates a significant difference; the bold values mean p value is less than 0.05*.

### The Role of *GRB10* rs1800504 Mutation in Liver Lipid Metabolism

**Figure 3:**
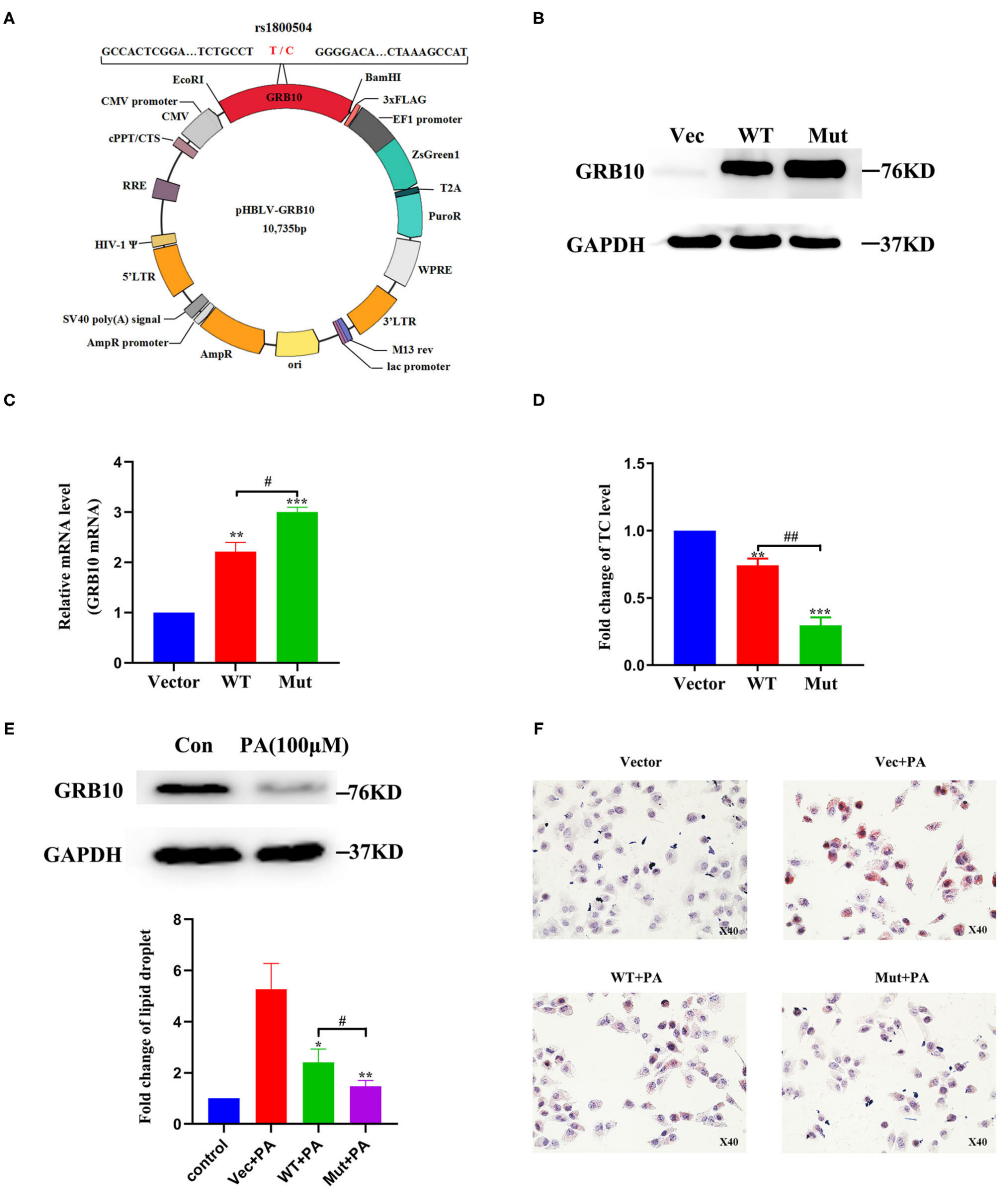
The role of *GRB10* rs1800504 mutation in MIHA cells. **(A)** Diagram showing the constructed vectors pHBLV-rs1800504-C and pHBLV-rs1800504-T. **(B)** GRB10 protein expression in MIHA cells, as determined by western blotting after the transfection of *GRB10* rs1800504 lentivirus. **(C)** GRB10 mRNA expression in MIHA cells assayed by q-PCR after transfection. **(D)** The levels of TC in MIHA cells transfected with *GRB10*-vector, *GRB10*-WT, and *GRB10*-Mut. **(E)** GRB10 expression in MIHA cells after 48 h of treatment with PA (100 μM). **(F)** Lipid droplet formation in MIHA cells transfected with *GRB10*-vector, *GRB10*-WT and *GRB10*-Mut after treatment with PA (100 μM) for 48 h. *p < 0.05, **p < 0.01, and ***p < 0.001 represent significantly differences compared with *GRB10*-vector. ^#^*p* < 0.05 and ^##^*p* < 0.01 represent significant differences compared with the *GRB10*-WT (*n* = 3).

To further determine the role of *GRB10* rs1800504 in liver lipid metabolism, we performed verification experiments *in vitro* with normal hepatocyte MIHA cells. We constructed a *GRB10* site-directed mutagenesis lentiviral vector and transfected the empty vector, *GRB10*-WT, and *GRB10*-Mut into MIHA cells (Figure 3A). We found that the expression of GRB10 in MIHA cells was significantly increased after transfection, and that the expression levels of cells transfected with *GRB10*-Mut were significantly higher than the levels of cells transfected with *GRB10*-WT (Figures 3B,C). Next, we measured the levels of TC in each group. Following the transfection of *GRB10*, the TC level in MIHA cells was significantly reduced, the levels of TC in the cells transfected with *GRB10*-Mut were 2–3 times lower than those in cells transfected with *GRB10*-WT (Figure 3D). Previous studies have shown that palmitic acid (PA) can significantly induce lipid accumulation in hepatocytes. We found that levels of the GRB10 protein were significantly decreased in MIHA cells that had been treated with PA (100 μM) (Figure 3E). After adding PA (100 μM) to MIHA cells transfected with *GRB10*-WT, *GRB10*-Mut, and the empty vector, we found that the overexpression of GRB10 reversed the formation of lipid droplets induced by PA, and we also discovered that the inhibitory effect of the *GRB10*-Mut vector had the most significant effect (Figure 3F).

## Discussion

For the first time, we found that *GRB10* gene polymorphism plays a role in the genetic susceptibility of T2DM-related CHD. Rs1800504 polymorphism of the *GRB10* gene was associated with the risk of CHD in T2DM patients. We also found that the genetic variation of rs1800504 was related to the levels of CHOL and LDL-CH. The levels of CHOL and LDL-CH in the mutated TT genotype were significantly lower than those in the CC+CT genotype. We found that the expression of GRB10 in MIHA cells transfected with *GRB10*-WT and *GRB10*-Mut were significantly different, thus indicating that mutation in the rs1800504 gene can affect the expression of GRB10. After the transfection of a GRB10 vectors, the levels of TC in MIHA cells were significantly reduced. The levels of TC in cells transfected with *GRB10*-Mut were 2–3 times lower than the levels in cells transfected with *GRB10*-WT. Using PA-induced lipid damage experiments, we found that *GRB10*-Mut was more effective than *GRB10*-WT in reversing the formation of lipid droplets induced by PA, thus confirming that rs1800504 genetic variation could result in different biological functions.

GRB10 is an adaptor protein that can interact with a variety of activated receptor protein tyrosine kinases, including the insulin receptor (IR), the insulin-like growth factor (IGF)-1 receptor, and the epidermal growth factor (EGF) receptor. A previous study showed that the overexpression of GRB10 in muscle cells and adipocytes inhibited insulin signaling, and that transgenic mice that overexpressed GRB10 showed impaired levels of glucose tolerance (25). It has also been reported that GRB10 is an important negative regulator of insulin/IGF-1 signaling in pancreatic β-cells, and it is also a potential target for improving β-cell function. As an inhibitor of insulin receptor signal transduction, GRB10 may become a candidate drug target for T2DM (26, 27). In one study, the minor allele (MA) of *GRB10* rs4947710 was associated with a reduced risk of T2DM in white subjects from Italy. In another SNP, *GRB10* rs2237457 was recently reported to be associated with T2DM in Amish subjects (15, 16). These results indicate that *GRB10* gene polymorphism may be closely related to susceptibility for diabetes. Previous analysis proved that *GRB10* is a key downstream mediator of VSMC miR-504 function. The upregulation of miR-504 in diabetes mellitus may be one of the mechanisms that enhances growth factor signals and VSMC dysfunction in vascular diseases. *GRB10* knockout can enhance the activation of ERK1/2 induced by PDGF, increase the expression of inflammatory genes (*CCL2* and *IL6*), and promote a pro-atherosclerotic phenotype in VSMC. The knockout of *GRB10* also enhanced VSMC migration while inhibiting EGR2 and contractile gene expression in a similar manner to miR-504 overexpression (19). These studies showed that *GRB10* is closely related to vascular diseases. Although *GRB10* is associated with both diabetes and vascular disease, the relationship between *GRB10* gene polymorphism and diabetic cardiovascular disease had not been studied previously. In this study, we genotyped 934 DNA samples from Chinese patients with T2DM, and found for the first time that *GRB10* rs1800504 genetic variation was associated with the occurrence of CHD in T2DM patients. Compared with the TT genotype, the CC+CT genotypes may be associated with a significant increase in CHD in T2DM patients (Table 3).

T2DM is often associated with hypercholesterolemia, dyslipidemia, hypertension, and obesity. Meanwile, these are also risk factors for cardiovascular events. The main cardiovascular event is atherosclerotic disease in patients with T2DM, while plasma lipids disorders are the basis of atherosclerosis (28–31). Therefore, the control of dyslipidemia has become critical goal for diabetic cardiovascular disease. In this study, we found that the levels of CHOL and LDL-CH in patients with the TT genotype of rs1800504 were significantly lower than those in patients with the CC+CT genotype (Figure 2 and Table 4). Therefore, allele C may represent as a risk factor for the abnormal elevation of CHOL and LDL-CH, thereby increasing the risk of CHD in T2DM patients. In recent years, an increasing evidence has indicated that the target of rapamycin complex (mTORC) plays a key role in the regulation of fat metabolism (32, 33). Two recent studies have shown that mTOR directly phosphorylates GRB10, and that cold exposure can significantly induce the expression of GRB10 in adipose tissue. In addition, the fat-specific knockout of GRB10 was shown to inhibit lipolysis and thermogenic gene expression, reduce energy consumption, and aggravate diet-induced obesity and insulin resistance. Collectively, these studies revealed that GRB10 is an important regulator of adipose tissue metabolism and energy homeostasis (34, 35). The liver is the main organ and for human lipid metabolism. Therefore hepatocyte MIHA cells were used as a model for *in vitro* experiments. When MIHA cells were transfected with *GRB10*-WT, *GRB10*-Mut, and *GRB10*-Vector, we found that the expression of GRB10 increased in cells transfected with the WT and Mut vectors, and that the expression levels of GRB10 in cells transfected with the Mut vector were significantly higher than cells transfected with the WT (Figures 3B,C). Western blotting found that when MIHA cells were treated with PA (100 μM) for 48 h, the levels of GRB10 protein had clearly decreased (Figure 3E). This result indicated that GRB10 may be related to plasma lipids metabolism. Moreover, both the *GRB10*-Mut and *GRB10*-WT vectors could reduce the levels of totalcholesterol (TC) and reverse the formation of lipid droplets induced by PA in MIHA cells (Figures 3D,F). Consistent with previous studies, our results indicate that *GRB10* rs1800504 mutation is a protective factor and can inhibit the excessive accumulation of cellular lipids. However, our conclusions need to be verified by further experiments and the mechanism of GRB10 regulating plasma lipids remains to be studied. Collectively, our data indicated that the influence of the rs1800504 mutation should be considered carefully in the diagnosis and intervention of T2DM complications.

## Conclusion

In conclusion, we report for the first time that *GRB10* rs1800504 genetic variation is closely related to the risk of CHD in T2DM patients. This mechanism may be achieved by regulating the levels of circulating blood lipids. Our *in vitro* experiments further confirmed that *GRB10* rs1800504 genetic variation is related to lipid metabolism in hepatocytes. However, our results need to be merited further study.

## Data Availability Statement

The datasets presented in this study can be found in online repositories. The names of the repository/repositories and accession number(s) can be found in the article.

## Ethics Statement

The studies involving human participants were reviewed and approved by Institute of Clinical Pharmacology, Central South University. The patients/participants provided their written informed consent to participate in this study.

## Author Contributions

YY: analyzed data and wrote manuscript. WQ: performed cell experiment and reviewed manuscript. QM: contributed to data reduction. WL and ML: collected the clinical samples. HY, RW, JD, and NY: recorded the clinical patient information. All authors contributed to the article and approved the submitted version.

## Funding

This study was supported by the Zhuhai People's Hospital (Zhuhai hospital affiliated with Jinan University) Cultivation project (No. 2009PY-09) and National Scientific Foundation of China (No. 81903715).

## Conflict of Interest

The authors declare that the research was conducted in the absence of any commercial or financial relationships that could be construed as a potential conflict of interest.

## Publisher's Note

All claims expressed in this article are solely those of the authors and do not necessarily represent those of their affiliated organizations, or those of the publisher, the editors and the reviewers. Any product that may be evaluated in this article, or claim that may be made by its manufacturer, is not guaranteed or endorsed by the publisher.
