# Supplementary material for: GRB10 rs1800504 Polymorphism Is Associated With the Risk of Coronary Heart Disease in Patients With Type 2 Diabetes Mellitus
Source: Front Cardiovasc Med. 2021 Sep 28;8:728976. doi: 10.3389/fcvm.2021.728976 (PMC8505721; doi:10.3389/fcvm.2021.728976)
Supplement: Supplementary file 1 [file Data_Sheet_1.doc]

**Supplementary files 1**

**
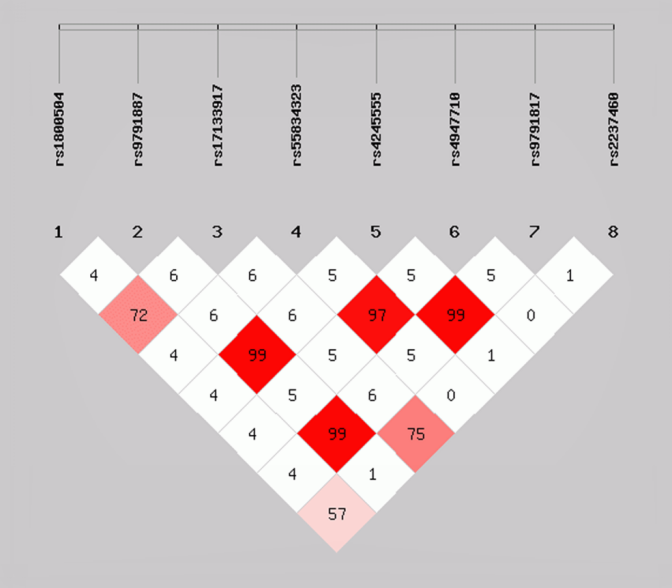
**

**Figure 1.The SNP linkage disequilibrium of *GRB10***

**Table 1. Information relating to Chip samples**

|  | | | | | |
| --- | --- | --- | --- | --- | --- |
| ID | Disease Classification | Chip Type | Standardization | Case Sample（n） | Normal Sample（n） |
| [GSE22255](https://www.ncbi.nlm.nih.gov/geo/query/acc.cgi?acc=GSE22255) | Ischemic Stroke | HG-U133_Plus_2 | RMA | 20 | 20 |
| [GSE58294](https://www.ncbi.nlm.nih.gov/geo/query/acc.cgi?acc=GSE58294) | Cardio-embolic Stroke | HG-U133_Plus_2 | -- | 69 | 23 |

**Table 2. Probe information for *GRB10* SNPs**

| SNP_ID | Probe sequences |
| --- | --- |
| rs1800504 | F:ACGTTGGATGTACCTGACAGCGAGGATGTG |
|  | R:ACGTTGGATGTCGGTCCATCCAGCCACAG |
| rs9791887 | F:ACGTTGGATGGAGTTGGCTGAGGTCGTATA |
|  | R:ACGTTGGATGCTTGTAAGTACCCACTGCAC |
| rs4947710 | F:ACGTTGGATGAAACAGGACGCGTGATAGAG |
|  | R:ACGTTGGATGGGCCTTACCCTCCAGGCGT |
| rs2237460 | F:ACGTTGGATGCCACCACTTCACATTTCTGC |
|  | R:ACGTTGGATGACCAGAGTTGGGCTTGTCAC |
| rs17133917 | F:ACGTTGGATGTCTATGTAGAGCCTCTGTCG |
|  | R:ACGTTGGATGCGGCAGAGTACCAGGAAGT |
| rs55834323 | F:ACGTTGGATGACATTCTAGTCCTCCCTGAG |
|  | R:ACGTTGGATGACCACATGGTCCAAATGCTG |
| rs4245555 | F:ACGTTGGATGCCCAGAGAGAATCATATTCC |
|  | R:ACGTTGGATGACAACACGACCTCCTTTCAG |
| rs9791817 | F:ACGTTGGATGTCATGGAGCAGAGTAAACCC |
|  | R:ACGTTGGATGGAGAAGACTGCACAAACCTG |

**Table 3. Predicted common SNPs with potential functional in the *GRB10* gene by ENCODE bioinformatics tools**

| Gene | SNP_ID | Chr | Function | MAF^a^ | MAF^b^ | Pb-HWD |
| --- | --- | --- | --- | --- | --- | --- |
| *GRB10* | rs1800504 | Chr7:50742080 | Synonymous variant | 0.43 | 0.40 | 0.16 |
|  | rs9791887 | Chr7:50661070 | Intron variant | 0.44 | 0.45 | **0.007** |
|  | rs4947710 | Chr7:50672779 | Synonymous variant | 0.08 | 0.06 | 0.24 |
|  | rs9791817 | Chr7:50593490 | Intron variant | 0.43 | 0.45 | **0.008** |
|  | rs2237460 | Chr7:50729261 | Missense variant | 0.46 | 0.49 | 0.59 |
|  | rs17133917 | Chr7:50709550 | Intron variant | 0.48 | 0.46 | 0.25 |
|  | rs55834323 | Chr7:50673255 | Intron variant | 0.08 | 0.06 | 0.25 |
|  | rs4245555 | Chr7:50661159 | Intron variant | 0.44 | 0.46 | **0.004** |

^a^ data from our study cohort (screening patients or all patients)

^b^ data from the1,000 Genomes Project (CHB)

SNP, single nucleotide polymorphism; GRB10, recombinant growth factor receptor bound protein 10; MAF, minimum allele frequency; HWD, Hardy-Weinberg disequilibrium. *P*＜0.05 indicates that the sample did not achieve genetic balance

**Table 4. Associations between *GRB10* candidate SNPs and the risk of CHD**

| RsID | Genotype | Number (%) | OR (95%CI) | *P* value |
| --- | --- | --- | --- | --- |
| 2237460 | CC | 271 (28.8) | Ref. | / |
|  | CT | 471 (50.1) | 1.59 (1.07-2.37) | 0.022 |
|  | TT | 192 (20.4) | 1.01 (0.61-1.69) | 0.96 |
| 17133917 | AA | 244 (26.0) | Ref. | / |
|  | AG | 481 (51.2) | 1.18 (0.78-1.77) | 0.43 |
|  | GG | 204 (21.7) | 0.70 (0.42-1.16) | 0.16 |
| 55834323 | CC | 788 (83.8) | Ref. | / |
|  | CT | 134 (14.3) | 1.30 (0.81-2.09) | 0.27 |
|  | TT | 9 (1.0) | 0.49 (0.05-4.43) | 0.52 |
| 4947710 | CC | 788 (83.8) | Ref. | / |
|  | CT | 133 (14.1) | 1.24 (0.77-1.99) | 0.38 |
|  | TT | 9 (1.0) | 1.85 (0.31-10.9) | 0.50 |
| 4245555 | TT | 272 (28.9) | Ref. | / |
|  | CT | 501 (53.3) | 0.97 (0.66-1.43) | 0.88 |
|  | CC | 155 (16.5) | 0.91 (0.54-1.53) | 0.72 |
| 9791817 | CC | 277 (29.5) | Ref. | / |
|  | GC | 500 (53.2) | 0.99 (0.68-1.46) | 0.96 |
|  | GG | 157 (16.7) | 0.80 (0.54-1.50) | 0.68 |
| 9791887 | AA | 274 (29.1) | Ref. | / |
|  | AC | 499 (53.1) | 0.99 (0.67-1.45) | 0.94 |
|  | CC | 157 (16.7) | 0.89 (0.53-1.49) | 0.89 |

CHD: [coronary heart disease](http://dict.youdao.com/w/coronary heart disease/" \l "keyfrom=E2Ctranslation). *p*＜0.05 indicates a significant statistical difference

***Table 5. Haplotype analysis results of the SNPs in GRB10***

| Loci | Case(freq) | Control(freq) | Chi2 | Fisher'sp | Pearson'sp | OddsRatio[95%CI] |
| --- | --- | --- | --- | --- | --- | --- |
| CAACCCCT | 0.00 (0.000) | 1.00 (0.001) | - | - | - | - |
| CAACTCCC | 8.20 (0.017) | 18.64 (0.013) | - | - | - | - |
| CAACTCCT* | 139.85 (0.282) | 358.83 (0.249) | 1.41 | 0.24 | 0.24 | 1.15 [0.91~1.45] |
| CAATTTCC* | 26.80 (0.054) | 69.76 (0.048) | 0.15 | 0.70 | 0.70 | 1.10 [0.69~1.73] |
| CAATTTCT | 11.84 (0.024) | 37.22 (0.026) | - | - | - | - |
| CAGCTCCC | 11.80 (0.024) | 38.38 (0.027) | - | - | - | - |
| CCACCCGT* | 75.14 (0.151) | 234.55 (0.162) | 0.63 | 0.43 | 0.43 | 0.89 [0.67~1.18] |
| CCACTCGT | 0.00 (0.000) | 1.00 (0.001) | - | - | - | - |
| CCATCCGT | 0.00 (0.000) | 1.09 (0.001) | - | - | - | - |
| CCATCTGC | 0.00 (0.000) | 0.24 (0.000) | - | - | - | - |
| CCATCTGT | 0.08 (0.000) | 1.36 (0.001) | - | - | - | - |
| CCGCCCGC* | 22.01 (0.044) | 42.72 (0.030) | 2.20 | 0.14 | 0.14 | 1.49 [0.88~2.51] |
| CCGCCCGT | 0.00 (0.000) | 2.20 (0.002) | - | - | - | - |
| CCGCTCCT | 0.00 (0.000) | 1.00 (0.001) | - | - | - | - |
| TAACTCCC | 0.00 (0.000) | 3.31 (0.002) | - | - | - | - |
| TAACTCCT | 1.10 (0.002) | 10.02 (0.007) | - | - | - | - |
| TAATTTCC | 0.00 (0.000) | 3.62 (0.003) | - | - | - | - |
| TAGCTCCC* | 76.23 (0.154) | 260.33 (0.180) | 2.518 | 0.11 | 0.11 | 0.80 [0.60~1.06] |
| TAGCTCCT | 0.00 (0.000) | 1.10 (0.001) | - | - | - | - |
| TAGTTTCC | 0.00 (0.000) | 5.80 (0.004) | - | - | - | - |
| TCACCCGC | 0.00 (0.000) | 1.73 (0.001) | - | - | - | - |
| TCACCCGT | 0.00 (0.000) | 6.64 (0.005) | - | - | - | - |
| TCGCCCCC | 0.00 (0.000) | 1.00 (0.001) | - | - | - | - |
| TCGCCCGC* | 120.67(0.243) | 340.47 (0.236) | 0.00 | 0.95 | 0.95 | 1.01[0.79~1.28] |
| TCGCCCGT | 0.00 (0.000) | 0.08 (0.000) | - | - | - | - |
| TCGCCTGC | 1.00 (0.002) | 1.00 (0.001) | - | - | - | - |
| TCGTCCGT | 0.00 (0.000) | 0.90 (0.001) | - | - | - | - |
| CAGTTTCC | 1.18 (0.002) | 0.00 (0.000) | - | - | - | - |
| CCGTCTGC | 0.11 (0.000) | 0.00 (0.000) | - | - | - | - |

**Supplementary files 2**

1. ***GRB10* sequence information**

atggctttagccggctgcccagattcctttttgcaccatccgtactaccaggacaaggtggagcagacacctcgcagtcaacaagacccggcaggaccaggactccccgcacagtctgaccgacttgcgaatcaccaggaggatgatgtggacctggaagccctggtgaacgatatgaatgcatccctggagagcctgtactcggcctgcagcatgcagtcagacacggtgcccctcctgcagaatggccagcatgcccgcagccagcctcgggcttcaggccctcctcggtccatccagccacaggtgtccccAaggcagagggtgcagcgctcccagcctgtgcacatcctcgctgtcaggcgccttcaggaggaagaccagcagtttagaacctcatctctgccggccatccccaatccttttcctgaactctgtggccctgggagcccccctgtgctcacgccgggttctttacctccgagccaggccgccgcaaagcaggatgttaaagtctttagtgaagatgggacaagcaaagtggtggagattctagcagacatgacagccagagacctgtgccaattgctggtttacaaaagtcactgtgtggatgacaacagctggacactagtggagcaccacccgcacctaggattagagaggtgcttggaagaccatgagctggtggtccaggtggagagtaccatggccagtgagagtaaatttctattcaggaagaattacgcaaaatacgagttctttaaaaatcccatgaatttcttcccagaacagatggttacttggtgccagcagtcaaatggcagtcaaacccagcttttgcagaattttctgaactccagtagttgtcctgaaattcaagggtttttgcatgtgaaagagctgggaaagaaatcatggaaaaagctgtatgtgtgtttgcggagatctggcctttattgctccaccaagggaacttcaaaggaacccagacacctgcagctgctggccgacctggaggacagcaacatcttctccctgatcgctggcaggaagcagtacaacgcccctacagaccacgggctctgcataaagccaaacaaagtcaggaatgaaactaaagagctgaggttgctctgtgcagaggacgagcaaaccaggacgtgctggatgacagcgttcagactcctcaagtatggaatgctcctttaccagaattaccgaatccctcagcagaggaaggccttgctgtccccgttctcgacgccagtgcgcagtgtctccgagaactccctcgtggcaatggatttttctgggcaaacaggacgcgtgatagagaatccggcagaggcccagagcgcagccctggaggagggccacgcctggaggaagcgaagcacacggatgaacatcctaggtagccaaagtcccctccacccttctaccctaagtacagtgattcacaggacacagcactggtttcacgggaggatctccagggaggaatcccacaggatcattaaacagcaagggctcgtggatgggctttttctcctccgtgacagccagagtaatccaaaggcatttgtactcacactgtgtcatcaccagaaaattaaaaatttccagatcttaccttgcgaggacgacgggcagacgttcttcagcctagatgacgggaacaccaaattctctgacctgatccagctggttgacttttaccagctgaacaaaggagtcctgccttgcaaactcaagcaccactgcatccgagtggccttatga

1. **Primer design**

| LV-h-GRB10Mutation-E/B-F | tactagaggatctatttccggtGaattcGCCACCatggctttagccggc |
| --- | --- |
| LV-h-GRB10Mutation-r | caccctctgcctTggggacacctgtggctgga |
| LV-h-GRB10Mutation-f | caggtgtccccAaggcagagggtgcagcgctcc |
| LV-h-GRB10Mutation-E/B-R | AGTCACTTAAGCTTGGTACCGAggatcctaaggccactcggatgcagtg |

1. **[S](http://dict.youdao.com/w/sequencing analysis/" \l "keyfrom=E2Ctranslation)equencing results**

TATTTGTCGTCATCATCCTTATAGTCCTTATCATCGTCGTCTTTGTAATCCTTGTCATCGTCATCCTTGTAGTCACTTAAGCTTGGTACCGAGGATCCTAAGGCCACTCGGATGCAGTGGTGCTTGAGTTTGCAAGGCAGGACTCCTTTGTTCAGCTGGTAAAAGTCAACCAGCTGGATCAGGTCAGAGAATTTGGTGTTCCCGTCATCTAGGCTGAAGAACGTCTGCCCGTCGTCCTCGCAAGGTAAGATCTGGAAATTTTTAATTTTCTGGTGATGACACAGTGTGAGTACAAATGCCTTTGGATTACTCTGGCTGTCACGGAGGAGAAAAAGCCCATCCACGAGCCCTTGCTGTTTAATGATCCTGTGGGATTCCTCCCTGGAGATCCTCCCGTGAAACCAGTGCTGTGTCCTGTGAATCACTGTACTTAGGGTAGAAGGGTGGAGGGGACTTTGGCTACCTAGGATGTTCATCCGTGTGCTTCGCTTCCTCCAGGCGTGGCCCTCCTCCAGGGCTGCGCTCTGGGCCTCTGCCGGATTCTCTATCACGCGTCCTGTTTGCCCAGAAAAATCCATTGCCACGAGGGAGTTCTCGGAGACACTGCGCACTGGCGTCGAGAACGGGGACAGCAAGGCCTTCCTCTGCTGAGGGATTCGGTAATTCTGGTAAAGGAGCATTCCATACTTGAGGAGTCTGAACGCTGTCATCCAGCACGTCCTGGTTTGCTCGTCCTCTGCACAGAGCAACCTCAGCTCTTTAGTTTCATTCCTGACTTTGTTTGGCTTTATGCAGAGCCCGTGGTCTGTAGGGGCGTTGTACTGCTTCCTGCCAGCGATCAGGGAGAAGATGTTGCTGTCCTCCAGGTCGGCCAGCAGCTGCAGGTGTCTGGGTTCCTTTGAAGTTCCCTTGGTGGAGCAATAAAGGCCAGATCTCCGCAAACACACATACAGCTTTTTCCATGATTTCTTTCCCAGCTCTTTCACATGCAAAAACCCTTGAATTTCAGGACAACTACTGGAGTTCAGAAAATTCTGCAAAAGCTGGGTTTGACTGCCATTTGACTGCTGGCACCAAGTAACCATCTGTTCTGGGAAGAAATTCATGGGATTTTTAAAGAACTCGTATTTTGCGTAATTCTTCCTGAATAGAAATTTACTCTCACTGGCCATGGTACTCTCCACCTGGACCACCAGCTCATGGTCTTCCAAGCACCTCTCTAATCCTAGGTGCGGGTGGTGCTCCACTAGTGTCCAGCTGTTGTCATCCACACAGTGACTTTTGTAAACCAGCAATTGGCACAGGTCTCTGGCTGTCATGTCTGCTAGAATCTCCACCACTTTGCTTGTCCCATCTTCACTAAAGACTTTAACATCCTGCTTTGCGGCGGCCTGGCTCGGAGGTAAAGAACCCGGCGTGAGCACAGGGGGGCTCCCAGGGCCACAGAGTTCAGGAAAAGGATTGGGGATGGCCGGCAGAGATGAGGTTCTAAACTGCTGGTCTTCCTCCTGAAGGCGCCTGACAGCGAGGATGTGCACAGGCTGGGAGCGCTGCACCCTCTGCCTTGGGGACACCTGTGGCTGGATGGACCGAGGAGGGCCTGAAGCCCGAGGCTGGCTGCGGGCATGCTGGCCATTCTGCAGGAGGGGCACCGTGTCTGACTGCATGCTGCAGGCCGAGTACAGGCTCTCCAGGGATGCATTCATATCGTTCACCAGGGCTTCCAGGTCCACATCATCCTCCTGGTGATTCGCAAGTCGGTCAGACTGTGCGGGGAGTCCTGGTCCTGCCGGGTCTTGTTGACTGCGAGGTGTCTGCTCCACCTTGTCCTGGTAGTACGGATGGTGCAAAAAGGAATCTGGGCAGCCGGCTAAAGCCATGGTGGCGAATTCACCGGAAATAGATCCTCTAGTAGAGTCGGTGTCTTCTATGGAGGTCAAAACAGCGTGGATGGCGTCTCCAGGCGATCTGACGGTCACTAAACGAGCTC
